# Efficacy of Lamotrigine in the Treatment of Unipolar and Bipolar Depression: Meta-Analysis of Acute and Maintenance Randomised Controlled Trials

**DOI:** 10.3390/ph18101590

**Published:** 2025-10-21

**Authors:** Danilo Arnone, Linda Östlundh, Meena Mosa, Brianne MacDonald, Jonathan Oldershaw, Tarik Qassem, Allan H. Young

**Affiliations:** 1 Centre for Affective Disorders, Psychological Medicine, Institute of Psychiatry, King’s College London, London SE5 8AF, UK; 2 College of Medicine, Mohammed Bin Rashid University of Medicine and Health Sciences, Dubai 505055, United Arab Emirates; 3 Division of Psychiatry, Department of Brain Sciences, Imperial College London, Burlington Danes, The Hammersmith Hospital, Du Cane Road, London W12 0NN, UK; 4 University Library, Örebro University, SE-702 81 Örebro, Sweden; 5 Faculty of Health Sciences, University of Ottawa, Ottawa, ON K1N 6N5, Canada; 6 Department of Psychology and Neuroscience, Dalhousie University, Halifax, NS B3H 4R2, Canada; 7 Royal Free London NHS Foundation Trust, London NW3 2QG, UK; 8 Maudsley Health, 201–205 Al-Montazah Tower, Khalidiya, Abu Dhabi 20002, United Arab Emirates; 9 Al Amal Psychiatric Hospital, Emirates Health Service, Dubai 50262, United Arab Emirates; 10 South London and Maudsley NHS Foundation Trust, London SE5 8AZ, UK

**Keywords:** depression, unipolar disorder, bipolar disorder, bipolar, major depressive disorders, lamotrigine, mood stabilisers

## Abstract

**Background/Objectives:** Lamotrigine has been widely investigated in the treatment and prevention of the emergence of symptoms of depression in unipolar and bipolar depression. This work systematically appraises published and unpublished double-blind randomised controlled trials of lamotrigine to provide up-to-date guidance on the use of lamotrigine in the presence of depressive symptoms. **Methods:** Systematic searches identified 32 randomised controlled trials, of which 24 were included in the meta-analysis, involving 2257 patients and 2320 controls. **Results:** Evidence supports the use of lamotrigine in the acute phase of bipolar depression in monotherapy vs. placebo (SMD: 0.155; CI: 0.005–0.305) in the absence of significant heterogeneity and small study effects. In the prophylaxis of bipolar depression, lamotrigine reduced the risk of the emergence of depressive symptoms (RR: 0.78; CI: 0.63, 0.98) and prolonged the duration of symptoms survival (RR: 1.59; CI: 1.19, 2.11) compared to placebo, with no evidence of publication and small study bias. Lamotrigine was not found to be superior to lithium in the acute treatment and prophylaxis of bipolar depression. In the treatment of unipolar depressive episodes, with the inclusion in the analyses of three unpublished studies, lamotrigine was not superior to placebo in monotherapy and as adjunct treatment. There were no maintenance studies in unipolar depression. **Conclusions:** There is evidence supporting the use of lamotrigine in monotherapy as acute and prophylactic treatment of bipolar depression. Evidence of the use of lamotrigine in unipolar disorders is lacking. PROSPERO registration ID: CRD42025633709.

## 1. Introduction

Lamotrigine is an inhibitor of voltage-sensitive sodium channels, specifically licenced for partial seizures, primary generalised tonic–clonic seizures and Lennox–Gastaut syndrome. The repurposing of lamotrigine to treat major depressive episodes is largely driven by the limited effectiveness of available treatments, and, in bipolar disorder, by the risk of cycle acceleration when antidepressants are used. The Food and Drug Administration currently supports the use of lamotrigine in the maintenance of bipolar disorder type I. In Europe, it is approved for preventing symptom emergence in bipolar depression [1,2,3].

Lamotrigine has been investigated in the treatment of acute depressive symptoms in both unipolar and bipolar depression. A recent network meta-analysis identified lamotrigine among the treatment options for bipolar depression, together with olanzapine plus fluoxetine, quetiapine, olanzapine, lurasidone, lumateperone and cariprazine [4]. This is in agreement with current treatment guidelines [5,6]. In unipolar depression, the use of lamotrigine is less clear, and it is generally considered a third-line treatment [5,6].

Available meta-analyses to date suggest that, in the acute treatment of bipolar depression, lamotrigine is (1) effective in monotherapy when compared with placebo (SMD: 0.16, CI: 0.03–0.29; RR: 1.27, CI: 1.09, 1.47), with a number needed to treat of 11 [4,7]; (2) effective in combination with other synergistic agents (SMD: −0.30, CI: −0.51, −10); (3) not superior to active comparators (SMD: −0.28; CI: −1.06, 0.50) [8]. In unipolar depression, the evidence for the use of lamotrigine is less clear. A meta-analysis that combined bipolar acute depression studies with three studies looking at unipolar depression suggested that lamotrigine is an effective antidepressant treatment overall (SMD −0.15; CI: −0.27, −0.02), with similar effectiveness in unipolar and bipolar depression based on statistical equivalence, although not superior to an active comparator [9]. Goh and colleagues found lamotrigine effective as an add-on medication in treatment-resistant depression based on six trials from China [10]. There has not been a meta-analysis which has combined published data with unpublished data from lamotrigine randomised double-blind trials in unipolar depression (https://www.gsk-studyregister.com, accessed on 21 May 2025).

The purpose of this meta-analysis was therefore to (1) systematically review the efficacy of lamotrigine as an acute and maintenance treatment in unipolar and bipolar depression; (2) provide a definitive summary of its efficacy by including published and unpublished studies.

## 2. Material and Methods

### 2.1. Literature Search

**Figure 1:**
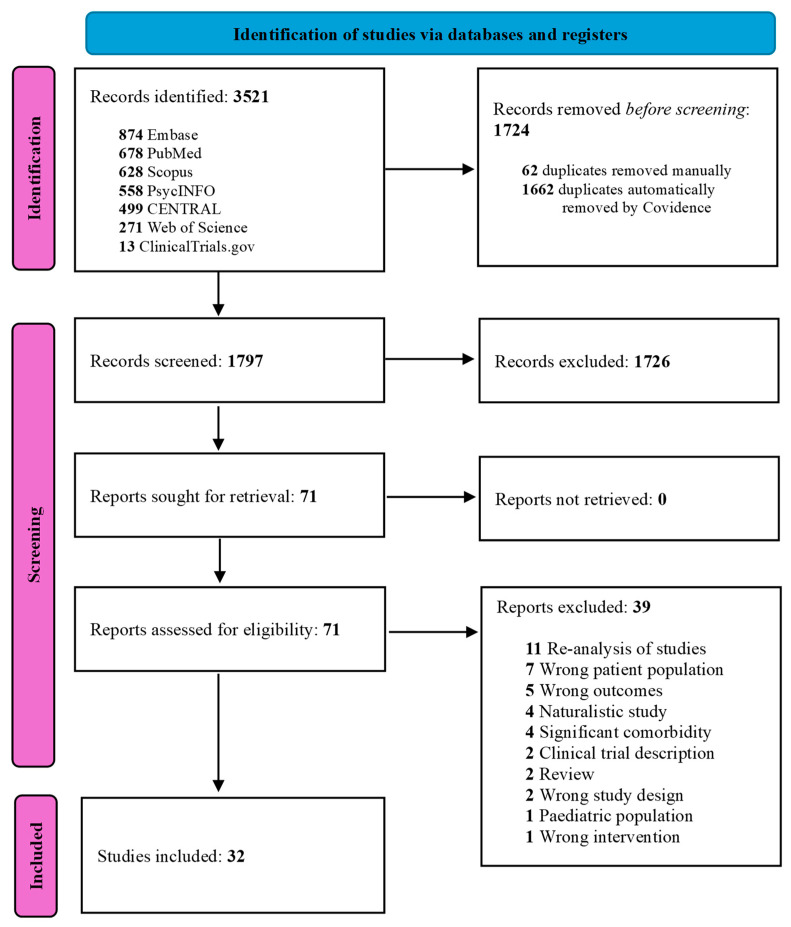
Prisma flow diagram [13].

A comprehensive literature search strategy was created (L.Ö.) and peer-reviewed (D.A.) to include studies from the databases inception up to June 2024. Biomedical databases, including PubMed, APA PsycINFO, Scopus, Web of Science, EMBASE, Cochrane Library, and the GSK study register (https://www.gsk-studyregister.com), were systematically searched. PubMed and PubMed’s MeSHs were used to systematically identify search term variations. A combination of the search fields ‘title’, ‘abstract’ and ‘MeSH/Thesaurus’ identified the best results. Key search terms included ‘Randomised Controlled Trial’, ‘Double Blind Controlled Trial’, ‘Bipolar depression’, ‘Major depression’, ‘Mood Disorders’, ‘Affective Disorders’, and ‘Lamotrigine’. All records were uploaded to the systematic review software Covidence (Veritas Health Innovation, Melbourne, Australia, 2020, https://www.covidence.org) for automatic de-duplication, and blinded screening by two independent reviewers (M.M. and B.M.) was performed. Selection discrepancies were resolved in the software by a third reviewer (D.A.). Identified papers that met the inclusion criteria were extracted and cross-referenced. The Preferred Reporting Items for Systematic Reviews and Meta-Analyses (PRISMA), PRISMA-S extension and Cochrane Handbook for Systematic Reviews of Interventions were employed for the search [11,12,13]. The results of the search and de-duplication were synthetised in a PRISMA flow-diagram [13] (Figure 1). PROSPERO registration was granted for this systematic review and meta-analysis (ID: CRD42025633709).

### 2.2. Eligibility Criteria, Data Extraction and Quality Assessment

The searches identified randomised trials that tested lamotrigine vs. placebo or other compounds in monotherapy or as adjuvant treatment for unipolar and bipolar depression diagnosed according to established diagnostic criteria. Double-blind randomised trials were included in the meta-analysis. The severity of depressive symptoms was evaluated by using validated rating scales. Studies that presented post hoc evaluations of data were excluded. In the case of multiple publications, the dataset with the largest sample size was considered. Studies with <10 participants were excluded (https://www.fda.gov/patients/drug-development-process/step-3-clinical-research, accessed on 2 September 2025). To maximise power and precision, changes in the rating scale score before and after the intervention were the preferred outcome measure (continuous outcome), as opposed to response/remission rates (binary outcome) [14]. The prioritization of continuous over binary clinical endpoints was based on evidence suggesting that binary outcome measures may lead to a loss of power [15]. In maintenance trials, outcomes included the rate of emergence of depressive symptoms and differences in survival time. The quality of the studies was screened independently by two assessors (J.O. and D.A.) by using the ‘Revised Cochrane risk-of-bias’ tool for randomised trials (RoB 2) [16]. A third author resolved conflicts by consensus (T.Q.).

### 2.3. Meta-Analysis

A random effect meta-analysis was conducted with STATA 18.0 (Stata Corp, College Station, TX, USA), supplemented by ‘Metan’ software v4.02 (David Fisher, MRC Clinical Trials Unit at UCL, London, UK), as previously described [17,18,19,20,21]. Studies were included if reported rating scale score changes as mean and standard deviation (SD), response/remission rates, rates of emergence of depressive symptoms and survival time. Risk ratios were calculated with STATA, and additional statistical analyses were performed according to conventional statistical methods [22,23]. Standardised mean differences were calculated using Cohen’s *d* statistic. Random effects analyses were used throughout to weight each study [24]. The effect of outliers was evaluated with the ‘leave one out method’ available in STATA. Sensitivity analyses were used to test the robustness of the results to test reasonable deviations from the assumption that missing data were a random occurrence [25]. The presence of heterogeneity was tested using the *Q*-test, with magnitude expressed with *I*^2^ representing the proportion of the effect size variance due to heterogeneity, where *I*^2^ values of 0.25, 0.50 and 0.75 were considered low, moderate and high, respectively [12,26]. Statistically significant heterogeneity was explored with meta-regressions. Confounders included year of publication, age, sex (number of women), number of participants with rapid cycling presentations, age at diagnosis, duration of episode, severity of depression, number of attempted suicides, and duration of the study. Egger’s test was used to examine small-study bias, alias the tendency of small studies to report large effect sizes with a significance level set at *p* ≤ 0.05 [27].

## 3. Results

Searches identified 3521 studies, of which 32 met the inclusion criteria (Figure 1). Twenty-four double-blind randomised control trials were included in the meta-analysis, including 2257 patients diagnosed according to DSM IV, DSM IV-TR and ICD-10 and 2320 controls. The quality of the studies was generally high, with a low or medium risk of bias. Details of the studies are presented in Table 1 and Table 2 and are described below.

### 3.1. Bipolar Disorder

#### 3.1.1. Acute Lamotrigine Monotherapy Treatment vs. Placebo

In this analysis, five studies that investigated lamotrigine vs. placebo in monotherapy were included [28,29,30,31,32]. Two studies could not be included because did not separate mood state from diagnosis in the outcome [50,51]. The studies included were generally high-quality randomised double-blind controlled trials that used an intention to treat analysis and last observation carried forward.

**Figure 2:**
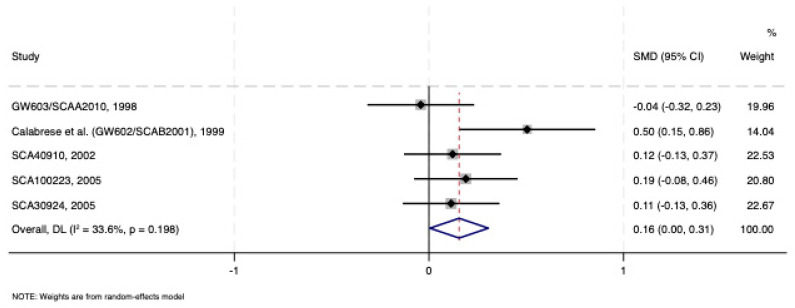
Forest plot of acute treatment studies in bipolar depression comparing lamotrigine vs. placebo in monotherapy [28,29,30,31,32]. See text for explanation.

Affected individuals were diagnosed with type I/II bipolar depression (N = 531, 314 women, mean age 40 years, no rapid cycling) and treated with lamotrigine 200–400 mg/day or a placebo (N = 516) for an average of 8.2 weeks in the randomised, double-blind studies. The severity of depressive symptoms was moderate to severe (98%); 35% attempted suicide and in 26% of cases the index episode lasted >24 weeks. A rash as a side effect occurred in 9% of cases in the lamotrigine-treated group. Lamotrigine was superior to placebo (SMD: 0.155; CI: 0.005–0.305). There was no evidence of heterogeneity (*I*^2^ = 33.6%, *p* = 0.198) or bias due to small-study bias (*p* = 0.152). There were no outliers that affected the results (Figure 2).

Lamotrigine was compared with lithium and olanzapine/fluoxetine in monotherapy in two randomised, controlled double-blind trials, suggesting comparable response/remissions rates. Suppes and colleagues compared lamotrigine (200 mg/day) with lithium in monotherapy in a 16-week single-blind randomised controlled trial. The two approaches were equally effective in the treatment of bipolar II depression, with comparable response and remission rates and no differences in rapid cyclers. Lamotrigine was better tolerated [33].

Brown and colleagues conducted two randomised double-blind studies comparing lamotrigine with olanzapine/fluoxetine [34,52]. The first 7-week acute study [52] was included in a subsequent publication which reported outcomes over a 6-month period [34]. Patients with bipolar depression received lamotrigine (200 mg/day) or olanzapine/fluoxetine combination in a randomised double-blind study. Olanzapine/fluoxetine (variable dose) was more effective in symptom reduction compared with lamotrigine, although response and remission rates were comparable [34].

#### 3.1.2. Acute Lamotrigine vs. Placebo as Adjunctive Treatment

Three double-blind randomised studies were included in the analysis which evaluated adjunctive lamotrigine or placebo to lithium [35], lithium and divalproate [36], and quetiapine [37]. Four studies were excluded due to the small number of participants [53,54,55,56]. The study by van Der Loos was excluded because it evaluated the effect of paroxetine in lamotrigine non-responders [57]. The study by Wang and colleagues was excluded because it evaluated co-morbid substance misuse disorder [58].

**Figure 3:**
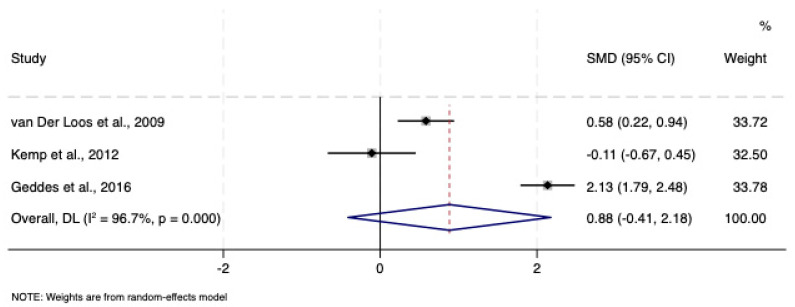
Forest plot of acute treatment studies in bipolar depression comparing adjunctive lamotrigine vs. adjunctive placebo [35,36,37]. See text for explanation.

In the three included studies, bipolar depression (N = 188, 106 women, mean age 40 years, 21% rapid cycling) was treated with lamotrigine 200–300 mg/day for an average of 10.6 weeks. The severity of depressive symptoms was largely moderate to severe. A rash was reported in four individuals (2%). There was a marginal clinical advantage of adding lamotrigine to mood stabilisers or quetiapine that did not reach statistical significance (N = 375; SMD: 0.88; CI: −0.41, 2.18). There was evidence of significant heterogeneity (*I*^2^ = 96.7%, *p* < 0.001), although none of the variables examined explained it (all ps > 0.005). There were no outliers and no evidence of bias due to small-study bias (*p* = 0.52) (Figure 3).

### 3.2. Maintenance Treatment

In prophylaxis, three datasets compared lamotrigine with placebo [38,39,40] and lamotrigine with lithium [39,40,41], in addition to the study by Brown and colleagues that compared lamotrigine with a combination of fluoxetine/olanzapine in variable dose over 6 months [34]. Three studies were not suitable because they evaluated the effect of paroxetine as an additional treatment to lamotrigine in non-responders [59], included co-morbid substance misuse disorder [58], and tested lamotrigine monotherapy vs. lamotrigine and divalproex [60].

#### 3.2.1. Lamotrigine vs. Placebo

In the lamotrigine (N = 365) vs. placebo (N = 276) comparison, patients were treated with lamotrigine (maximum dose 400–500 mg/day) for an average of 14 months (mean age 41 years old, 186 women, 32% rapid cyclers, 33 average number of suicide attempts). A rash was reported in 4.6% of cases.

**Figure 4:**
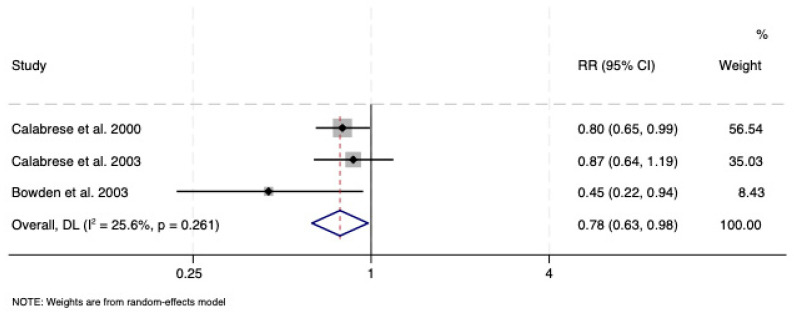
Forest plot of risk of symptom emergence of lamotrigine monotherapy vs. placebo in bipolar depression [38,39,40]. See text for explanation.

**Figure 5:**
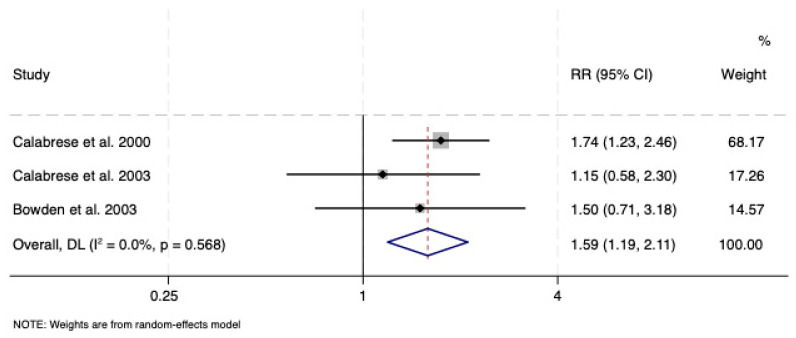
Forest plot of survival duration under lamotrigine vs. placebo as monotherapy in bipolar depression [38,39,40]. See text for explanation.

The risk of emergence of depressive symptoms was reduced with lamotrigine vs. placebo (RR: 0.78; CI: 0.63, 0.98), with no evidence of significant heterogeneity (*I*^2^ = 25.6%, *p* = 0.261) or publication bias (*p* = 0.42) (Figure 4). Lamotrigine was protective in relation to depressive symptom survival duration vs. placebo (RR: 1.59; CI: 1.19, 2.11), with no heterogeneity (*I*^2^ = 0.0%, *p* = 0.57) or small-study bias (*p* = 0.38). No outliers were noted (Figure 5).

#### 3.2.2. Lamotrigine vs. Lithium

In the lamotrigine (N = 350) vs. lithium (N = 242) comparison, patients were treated with lamotrigine (maximum dose 400 mg/day) for an average of 35 months (mean age 41 years old, 171 women, 13% rapid cyclers, 96 suicide attempts). A rash was reported in 5.7% of cases.

The risk of emergence of depressive symptoms was not statistically significant between lamotrigine and lithium (RR: 0.82; CI: 0.63, 1.06), with no small-study (*I*^2^ = 0.0%, *p* = 0.55) and publication bias (*p* = 0.16). There was no difference in depressive symptom survival duration between the two groups (RR: 0.90; CI: 0.68, 1.18), with no small study bias (*I*^2^ = 0.0%, *p* = 0.60) or publication bias (*p* = 0.56). No outliers were noted.

#### 3.2.3. Lamotrigine vs. Olanzapine/Fluoxetine Combination

Brown and colleagues investigated 205 patients with moderate to severe bipolar depression in each group. There was no difference in the emergence of depressive symptoms between patients treated with lamotrigine and olanzapine/fluoxetine combination. A rash occurred in 8.8% of lamotrigine-treated patients [34].

### 3.3. Unipolar Depression

#### 3.3.1. Acute Monotherapy Treatment

Four double-blind studies explored the efficacy of lamotrigine (N = 395) vs. placebo (N = 394) in monotherapy for an average of 7.5 weeks; three studies were unpublished [42,43,44] and one recruited treatment-resistant patients [45]. The mean age of individuals treated with lamotrigine was 40 years of age; 236 were women and 7.6% experienced a rash. Overall, there was no significant effect that distinguished lamotrigine from placebo (SMD: 0.10; CI: −0.20, 0.41) with no outliers. There was evidence of heterogeneity (*I*^2^ = 73.7%, *p* = 0.01) that was not explained by the variables examined (all ps > 0.05). There was no small-study bias (*p* = 0.39).

#### 3.3.2. Acute Adjunctive Treatment

Three studies evaluated lamotrigine vs. placebo as an additional treatment to selective serotonin reuptake inhibitors (SSRIs) fluoxetine [46] and paroxetine [47,48]. The outcome of the analysis which compared response rates suggested no significant benefit of lamotrigine added to SSRIs (RR: 0.95; CI: 0.73, 1.23), no outliers, no evidence of small-study bias (*I*^2^ = 0.0%, *p* = 0.45), and no publication bias (*p* = 0.26).

#### 3.3.3. Acute Comparison with an Active Compound

A randomised double-blind trial that compared lamotrigine with placebo also included a desipramine treatment arm. The analysis did not differentiate desipramine from lamotrigine or placebo [42]. Another randomised open-label study compared lamotrigine with lithium as an add-on to current treatment in patients with treatment-resistant depression. In this study, carried out by Schindler and Anghelescu, response and remission rates were similar in lamotrigine- and lithium-treated patients [49].

## 4. Discussion

In this meta-analysis, we set out to review the efficacy of lamotrigine in acute and maintenance studies of bipolar and unipolar depression.

In bipolar disorders, lamotrigine was more effective in the acute treatment of depression as monotherapy when compared with placebo. This result is consistent with Yildiz and colleagues’ (SMD: 0.16; CI: 0.03, 0.29) [4] and Geddes and colleagues’ (SMD: −0.12; 95% CI: −0.24, 0.00), based on the Montgomery Åsperg Depression Rating Scale (MÅDRS). Geddes and colleagues also reported higher pooled response (RR: 1.27, CI: 1.09, 1.47) and remission rates (RR: 1.21, CI: 1.03, 1.42) for lamotrigine vs. placebo and calculated an NNT = 11 for response rates [7].

There were only two randomised double-blind trials we could include that compared lamotrigine to an active compound including lithium and olanzapine/fluoxetine. Both trials suggested equivalence in response and remission rates in bipolar depression. This is consistent with previous published work [8]. Additionally, three randomised double-blind trials were included that tested the efficacy of lamotrigine added to existing pharmacological treatments for bipolar depression. In this case, lamotrigine was not found to be superior. This result differs from Haenen and colleagues. The authors suggested that lamotrigine is superior as an add-on treatment in bipolar disorder (SMD: −0.30, CI: −0.51, −10) [8]. The difference is most likely due to our selection criteria, which focused on double-blind randomised studies and excluded the study by Wang and colleagues that tested lamotrigine in bipolar depression and comorbid substance misuse disorder [58].

As prophylaxis, lamotrigine reduced the risk of emergence of depressive symptoms when compared to placebo (RR: 0.78; CI: 0.63, 0.98) and not lithium. This is consistent with previous, similar work focused on bipolar symptoms, leading to the same conclusion that lamotrigine reduces symptoms emergence only vs. placebo (RR 0.84; CI: 0.71, 0.99) [8]. Consistent with this notion, we additionally demonstrated that lamotrigine was superior to placebo in terms of depressive symptom survival duration, in that it extended the time to recurrence (RR: 1.59; CI: 1.19, 2.11).

The standardised mean difference for lamotrigine in the acute treatment of bipolar depression is equivalent to 0.155. Standardised mean values of 0.2, 0.5, and 0.8 are conventionally considered small, medium, and large effect sizes. This suggests that there is a large overlap between the distributions of lamotrigine vs. placebo that renders group differentiation difficult [61,62]. Clinically, this finding indicates low efficacy of lamotrigine in the acute phase of the treatment of bipolar depression. In prophylaxis, the effect of lamotrigine in reducing the risk of relapse of bipolar depression vs. placebo was more pronounced, based on an RR of 0.78, conferring 22% risk reduction. Similarly, the length of time without depressive symptoms, based on an RR of 1.59, suggested a 59% increased chance with lamotrigine.

In relation to unipolar depression, lamotrigine was not effective in the acute treatment of depressive symptoms. Previous work by Solmi and colleagues [9] concluded that lamotrigine was effective in the treatment of acute symptoms of unipolar depression based on tests of homogeneity between bipolar and unipolar depression of the combined pooled bipolar and unipolar data. However, their meta-analysis of the unipolar data suggested no significant effect in relation to studies comparing lamotrigine vs. placebo in monotherapy (SMD: −0.06; CI: −0.41, 0.28) and as dd-on treatment (RR: 1.13; CI: 0.80, 1.60). These observations agree with our findings.

As an adjunctive treatment in the acute phase of unipolar depression, our results suggest lack of efficacy. This contrasts the findings by Goh and colleagues from their research on treatment-resistant depression [10]. Their work included Chinese studies that were not available by using conventional searching methods.

A rash was reported in lamotrigine-treated patients. There were no noted occurrences of Steven Johnson syndrome.

Limitations of this work include the relatively small number of studies, leaving the possibility that small-study bias might have occurred, despite the absence of statistical significance in the analyses. Only randomised double-blind studies were selected for the meta-analysis, and quality assessments were generally positive. However, it is plausible that sampling bias could have played a confounding role in the analyses, especially given that significant heterogeneity could not be explained by the variables considered. The influence of confounders on the effect size was evaluated with meta-regression analyses. This method was chosen because it was not possible to run sub-analyses due to the inconsistent and limited data availability across the studies and the relatively small number of trials.

Advantages of including unpublished data in a systematic review include reducing the risk of publication bias, which is conducive to more accurate and precise estimates of treatment effects. Limitations potentially include data quality and the theoretical risk of introducing different biases due to the possible selective nature of the data [63,64].

We were not able to dichotomise outcomes according to sex because data was reported in the original papers at whole-group level. Measuring the effect size of lamotrigine in women could only have provided additional guidance to clinicians when balancing the pros/cons of prescribing during pregnancy and/or breastfeeding. Meta-regression analyses did not suggest sex effects in relation to outcome.

The duration of the original studies varied. Although this could have influenced the outcome of each study, meta-regression analyses suggested that it did not significantly influence the overall effect size.

The repurposing of lamotrigine to treat depressive symptoms in bipolar disorder is a good example of the successful, transdisciplinary use of pharmacology [65]. Future work focusing on the mechanisms of action of lamotrigine on emotional regulation at brain level could improve our understanding of brain function and aid the development of more effective treatments [66].

## 5. Conclusions

Lamotrigine is indicated in the treatment of bipolar depression to ameliorate acute symptoms, and, most importantly, in maintaining recovery. Analyses do not support the use of lamotrigine in unipolar depression.

## Figures and Tables

**Table 1:** Study details. HAM-D: Hamilton Depression Rating Scale, 17 or 21 items; MÅDRS: Montgomery Åsperg Depression Rating Scale; MES: Bech–Rafaelsen Melancholia Scale.

Study	Bipolar Type	Comparator	Lamotrigine Target Dose	Lamotrigine N.	Comparator N.	Mean Age	Women (Cases) N.	Duration Weeks	Rating Scale	Risk of Bias
Bipolar depression: Acute phase monotherapy										
Calabrese et al. (GW602/SCAB2001), 1999 [28]	I	Placebo	200	63	66	42	35	7	HDRS17/MÅDRS/CGI-S	Low
GW603/SCAA2010, 1998 [29]	I/II	Placebo	400	103	101	40.5	66	10	HDRS17/MÅDRS/CGI-S	Low
SCA40910, 2002 [30]	I	Placebo	200	129	118	37.6	74	8	HDRS17/MÅDRS/CGI-S	Low
SCA100223, 2005 [31]	II	Placebo	200	109	109	38.1	70	8	HDRS17/MÅDRS/CGI-S	Low
SCA30924, 2005 [32]	I	Placebo	200	127	122	40.5	69	8	HDRS17/MÅDRS/CGI-S	Low
Suppes et al., 2008 [33]	II	Lithium	400	41	49	36.9	30	16	HDRS17	Medium
Brown et al., 2009 [34]	I	Olanzapine + Fluoxetine	200	205	205	36.8	128	25	MÅDRS	Medium
				777	770	38.9	472	11.7		
Bipolar depression: Acute phase add-on								Weeks		
van Der Loos et al., 2009 [35]	I/II	Lithium + Placebo	200	64	60	45.2	37	8	MÅDRS	Low
Kemp et al., 2012 [36]	I/II	Lithium/Divalproate + Placebo	200	23	26	35.7	12	12	MÅDRS	Low
Geddes et al., 2016 [37]	I/II	Quetiapine + Placebo	300	101	101		57	12	QIDS SR 16	Medium
				188	187	40.45	106	10.6		
Bipolar depression: Maintenance phase								Months		
Calabrese et al. 2000 [38]	I/II	Placebo	500	92	88	38.5	55	6	HAM-D-17	Low
Calabrese et al., 2003 [39]	I	Placebo	500	215	119	44.1	99	18	HAM-D-17	Low
Calabrese et al., 2003 [39]	I	Lithium	500	NA	120	NA	NA	18	HAM-D-17	
Bowden et al., 2003 [40]	I	Placebo	400	58	69	40.6	32	18	HAM-D-17	Low
Bowden et al., 2003 [40]	I	Lithium	400	NA	44	NA	NA	18	HAM-D-17	
Brown et al., 2009 [34]	I	Olanzapine + Fluoxetine	200	205	205	36.8	128	25	MÅDRS	Medium
Licht et al., 2010 [41]	I	Lithium	400	77	78	38.2	40	69.59	MES	Medium
				647	723	39.64	354	24.6		
Unipolar depression: Acute phase monotherapy								Weeks		
SCAA2011, 1998 [42]	NA	Placebo	200	152	150	39	99	8	HAM-D 17/MÅDRS/CGI-I	Low
SCA20022, 1999 [43]	NA	Placebo	200	75	77	41.9	32	7	HAM-D 17/MÅDRS/CGI-I	Low
SCA20025, 2000 [44]	NA	Placebo	200	151	150	41.1	40	7	HAM-D 17/MÅDRS/CGI-I	Low
Santos et al., 2008 [45]	NA	Placebo	200	17	17	38.2	171	8	MÅDRS	Low
				395	394	40.05	342	7.5		
Unipolar depression: Acute phase add-on										
Barbosa et al., 2003 [46]	NA	Fluoxetine + Placebo	100	13	10	30.2	5	6	HAM-D-17	Medium
Normann et al., 2002 [47]	NA	Paroxetine + Placebo	200	20	20	39.6	14	9	HAM-D-21	Medium
Barbee et al., 2011 [48]	NA	Paroxetine + Placebo	400	48	48	44.59	33	10	HAM-D 17/MÅDRS	Low
				81	78	38.13	52	8.3		
Unipolar depression: Direct comparison								Weeks		
SCAA2011, 1998 [42]	NA	Desipramine	200	152	151	39	86	8	HAM-D 17/MÅDRS/CGI-I	Low
Schindler and Anghelescu, 2007 [49]	NA	Lithium	250	17	17	41.1	9	8	HAM-D 17	High
				169	168	40.05	95	8		

**Table 2:** Summary of efficacy of lamotrigine in unipolar and bipolar depression.

	Bipolar Depression	Unipolar Depression
Acute phase in monotherapy	Evidence of superior efficacy of lamotrigine vs. placebo but not vs. lithium or vs. olanzapine/fluoxetine combination	No superior efficacy of lamotrigine vs. placebo or vs. desipramine
Acute phase as add-on treatment	No superior efficacy of lamotrigine when added to lithium or valproate compared to placebo	No superior efficacy of lamotrigine when added to fluoxetine or paroxetine
Maintenance phase	Evidence of superior efficacy of lamotrigine vs. placebo but not vs. lithium or vs. olanzapine/fluoxetine combination	No available studies

## Data Availability

No new data were created or analyzed in this study.
